# Supplementary material for: RNA Sequencing and Weighted Gene Co-Expression Network Analysis Highlight DNA Replication and Key Genes in Nucleolin-Depleted Hepatoblastoma Cells
Source: Genes (Basel). 2024 Nov 26;15(12):1514. doi: 10.3390/genes15121514 (PMC11675179; doi:10.3390/genes15121514)
Supplement: Supplementary file 1 [file genes-15-01514-s001.zip › genes-3315717-supplementary.pdf]

Supplementary information

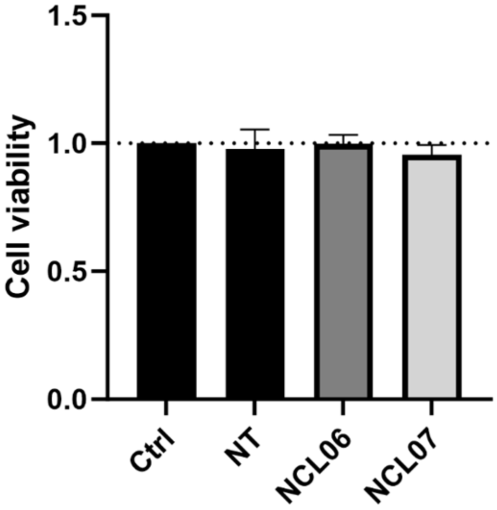

Supplementary Figure S1. Effects of siRNA-mediated NCL knockdown on cell viability. HepG2 cells were incubated with 10 nM of different siRNAs (NT, NCL06 and NCL07) for 72 hours. Untreated cells were used as control (Ctrl). Cell survival was determined using the EZ4U cell viability assay. Conditions were normalized to the corresponding wild type control (Ctrl). The data represents the mean values ± SDs of two biological replicates with three technical replicates each.

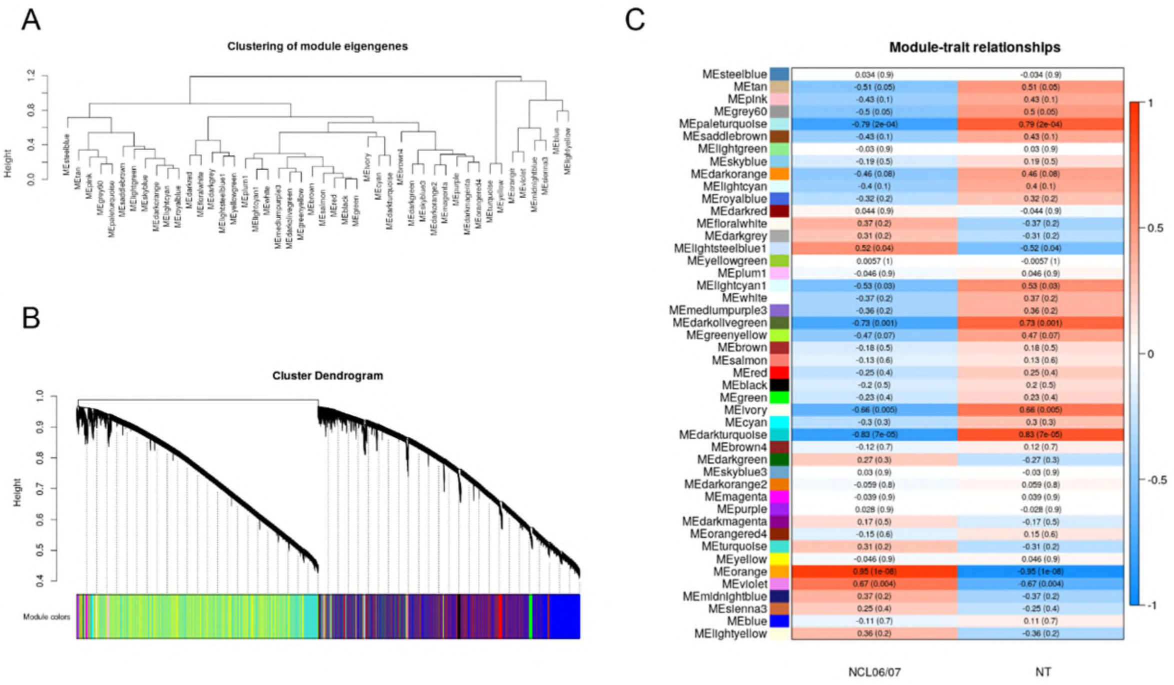

Supplementary Figure S2 A-C. Sample clustering and network construction with NCL06, NCL07 and NT siRNA treated HepG2 cell associations. (A) Clustering dendrogram of module eigengenes. **(B)** Dendrogram clustered based on a dissimilarity measure. **(C)** Heatmap of the correlation between module eigengenes and the NCL06 and NCL07 knockdown related genes. Each module contains the corresponding correlation value and *p*-value.

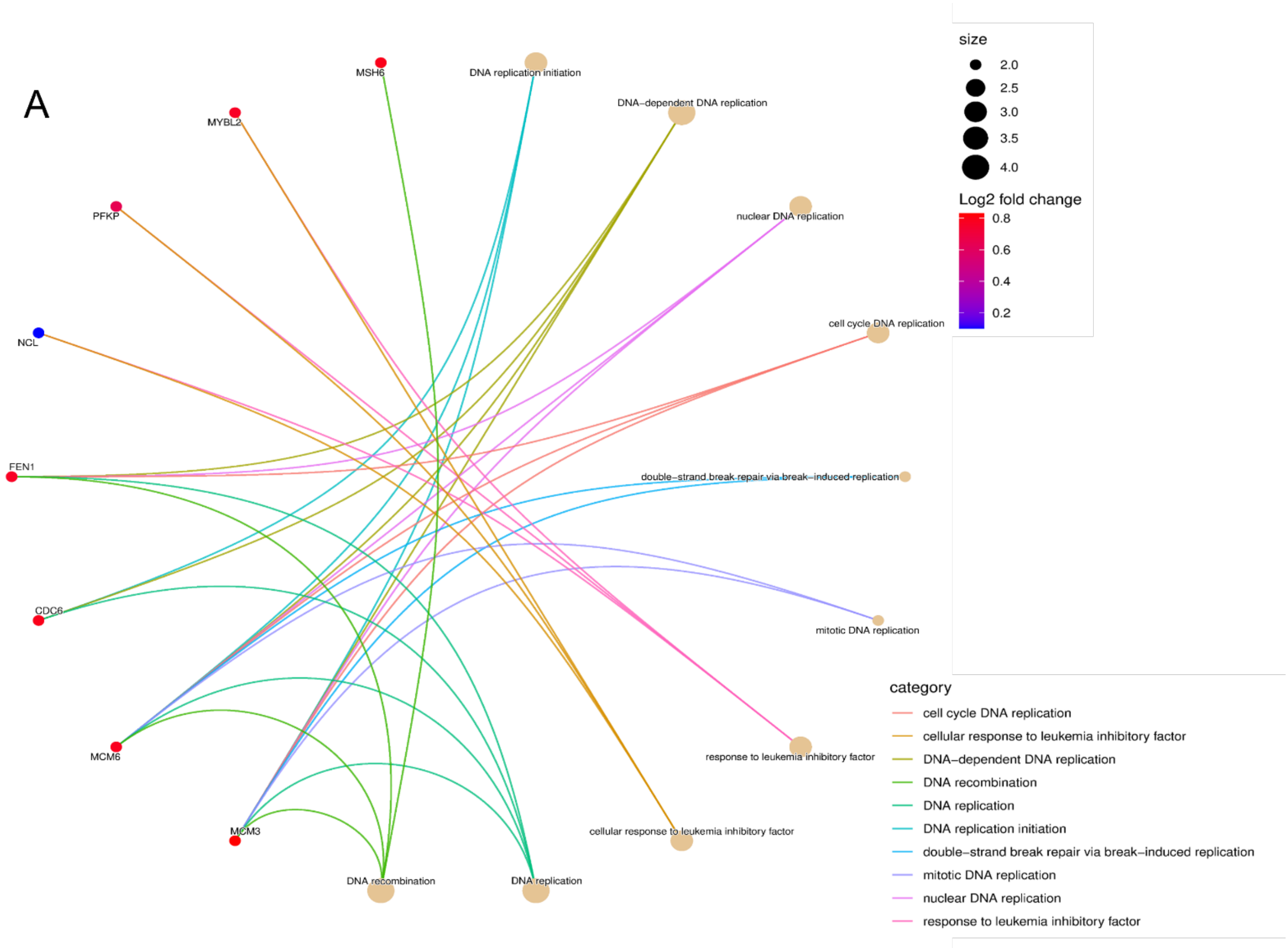
A
MSH6
MYBL2
PFKP
NCL
FEN1
CDC6
MCM6
MCM3
DNA recombination
DNA replication
cellular response to leukemia inhibitory factor
response to leukemia inhibitory factor
mitotic DNA replication
double−strand break repair via break−induced replication
cell cycle DNA replication
nuclear DNA replication
DNA−dependent DNA replication
DNA replication initiation
size
2.0
2.5
3.0
3.5
4.0
Log2 fold change
0.8
0.6
0.4
0.2
category
cell cycle DNA replication
cellular response to leukemia inhibitory factor
DNA−dependent DNA replication
DNA recombination
DNA replication
DNA replication initiation
double−strand break repair via break−induced replication
mitotic DNA replication
nuclear DNA replication
response to leukemia inhibitory factor

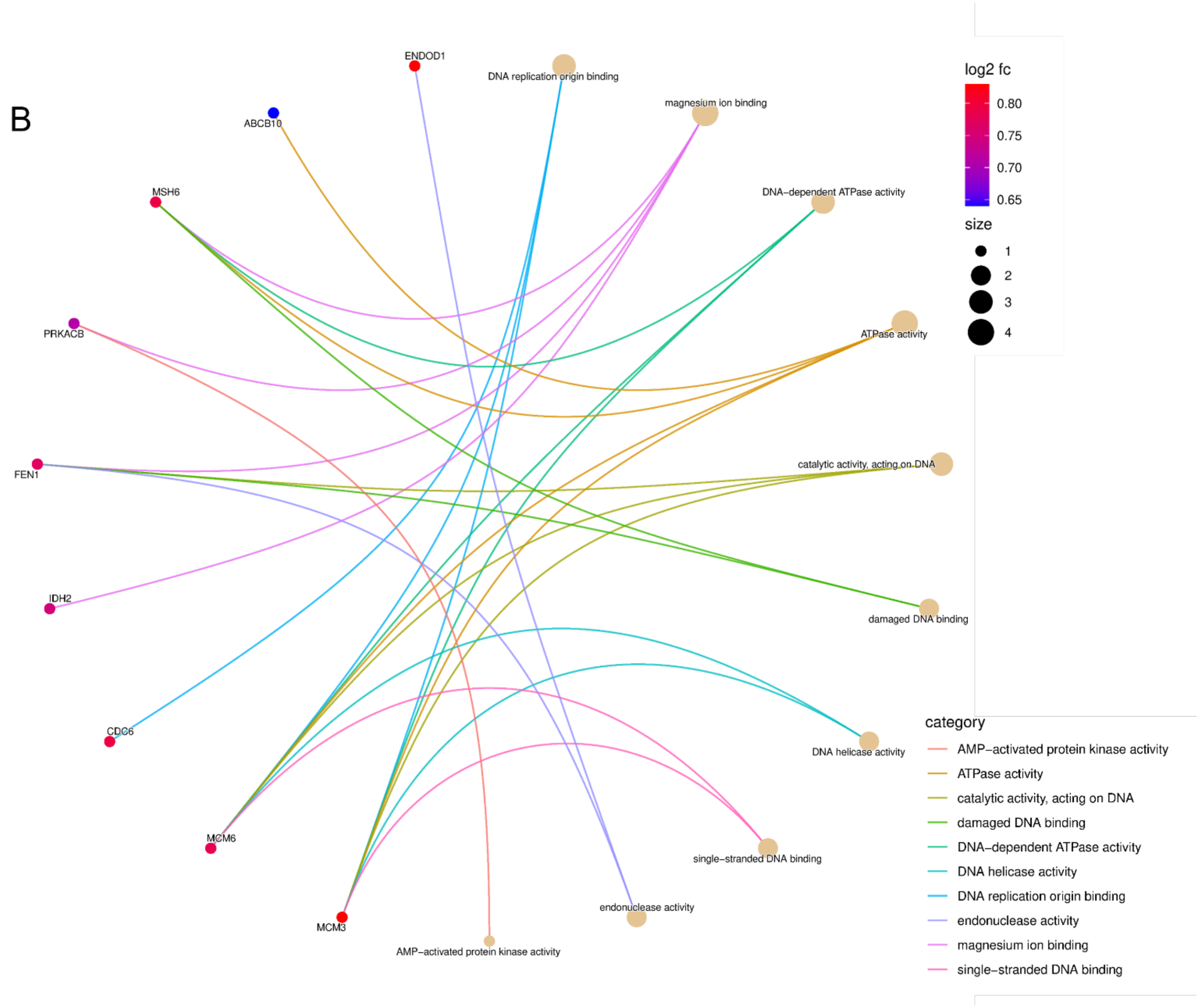

Supplementary Figure S3 A/B. cnet blot of biological processes (A) and molecular functions (B)

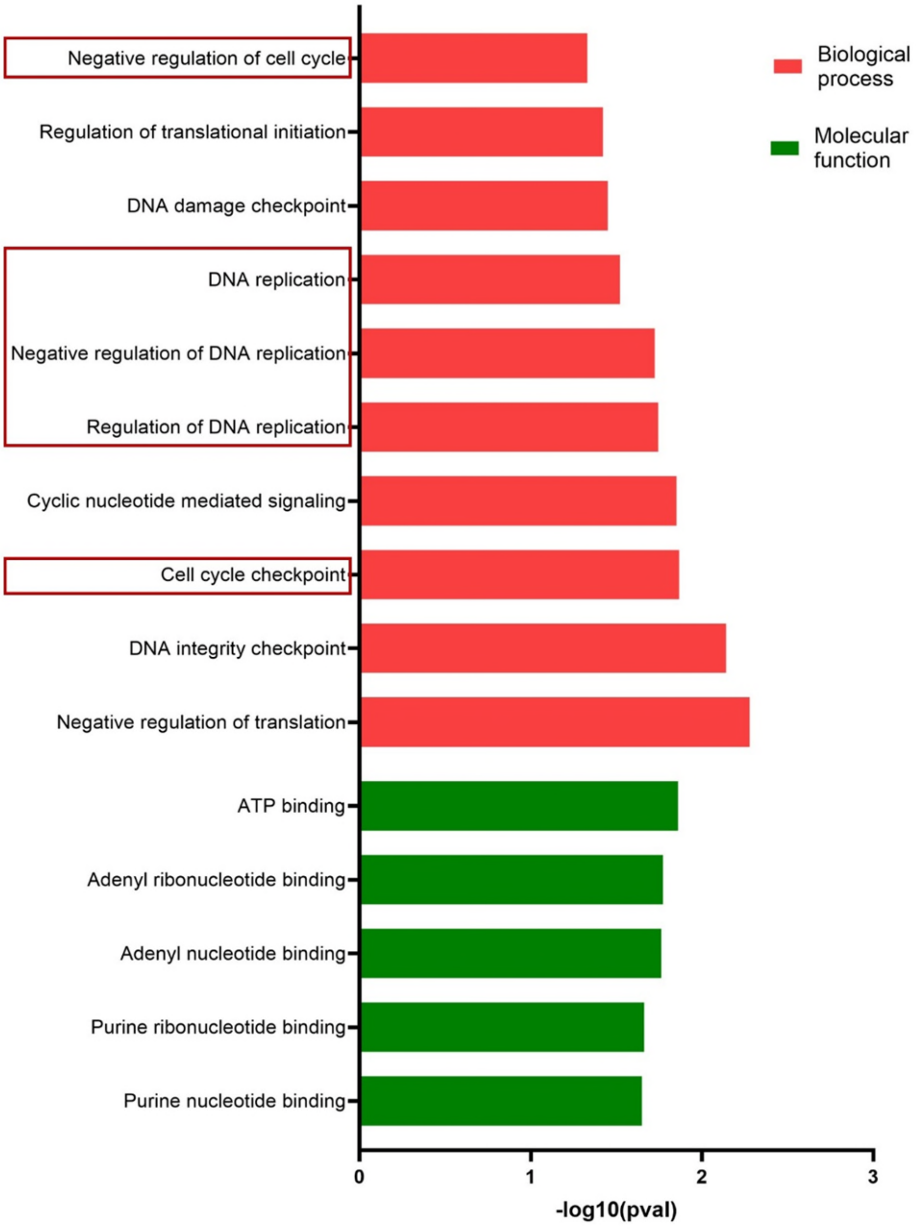

Supplementary Figure S4. Enrichment analysis with genes from WGCNA. Enrichment analysis of 72 approved genes from most significant modules: r > 0.7: orange (0.95), dark turquoise (−0.83), pale turquoise (−0.79) and dark olive green (−0.73). The red boxes represent pathways connected to DNA replication and cell cycle.

Supplementary Table S1. The 44 up- and downregulated DEGs (ranked by p-value) between the NCL06 and NCL07 versus NT knockdown groups

gene	pval	padj	log2FC	description
RBM14	1.2e-38	1.9e-34	1.11	RNA binding motif protein 14
NCL	3.7e-12	2.8e-08	-3.29	nucleolin
AQP3	3.1e-11	1.6e-07	0.79	aquaporin 3 (Gill blood group)
PSPC1	5.7e-10	2.2e-06	0.58	paraspeckle component 1
IDH2	7.6e-10	2.4e-06	-0.42	isocitrate dehydrogenase (NADP(+)) 2
DDX17	1.2e-09	3.1e-06	0.32	DEAD-box helicase 17
ABCB10	4.8e-09	1.1e-05	-0.65	ATP binding cassette subfamily B member 10
IBTK	7.4e-09	1.4e-05	-0.39	inhibitor of Bruton tyrosine kinase
UBE2G2	1.2e-08	2.0e-05	-0.45	ubiquitin conjugating enzyme E2 G2
GPR146	2.4e-08	3.7e-05	5.44	G protein-coupled receptor 146
DDC8	4.3e-08	6.1e-05	4.71	CEP295 N-terminal like
BTG2	9.1e-08	1.2e-04	0.64	BTG anti-proliferation factor 2
FEN1	1.1e-07	1.3e-04	-0.38	flap structure-specific endonuclease 1
PFKP	1.2e-07	1.4e-04	-0.65	phosphofructokinase, platelet
TEKT4P2	1.6e-07	1.7e-04	-0.79	tektin 4 pseudogene 2
HGSNAT	2.1e-07	2.0e-04	-0.68	heparan-alpha-glucosaminide N-acetyltransferase
MYEOV	2.2e-07	2.0e-04	0.67	myeloma overexpressed
CCDC107	3.4e-07	2.9e-04	0.62	coiled-coil domain containing 107
PI4K2B	3.7e-07	3.0e-04	-0.46	phosphatidylinositol 4-kinase type 2 beta
SCAMP4	4.8e-07	3.8e-04	-0.62	secretory carrier membrane protein 4
CASC10	1.6e-06	1.2e-03	0.73	cancer susceptibility 10
ST3GAL3	1.8e-06	1.3e-03	-0.83	ST3 beta-galactoside alpha-2,3-sialyltransferase 3
MCM3	2.4e-06	1.6e-03	-0.27	minichromosome maintenance complex component 3
PRKACB	4.0e-06	2.6e-03	-0.5	PRKACB divergent transcript
MYBL2	1.1e-05	6.6e-03	-0.38	MYB proto-oncogene like 2
CNDP2	1.8e-05	1.2e-02	-0.37	carnosine dipeptidase 2
RASD1	2.5e-05	1.4e-02	0.37	ras related dexamethasone induced 1
ROGDI	3.4e-05	1.8e-02	-0.37	rogdi atypical leucine zipper
ENDOD1	3.8e-05	2.0e-02	-0.27	endonuclease domain containing 1
AKR1C1	4.3e-05	2.2e-02	0.66	aldo-keto reductase family 1 member C1
MSH6	4.7e-05	2.3e-02	-0.34	mutS homolog 6
RBM4	5.1e-05	2.3e-02	0.62	RNA binding motif protein 4
MCM6	5.3e-05	2.3e-02	-0.35	minichromosome maintenance complex component 6
CDC6	5.4e-05	2.3e-02	-0.34	coiled-coil domain containing 6
NDRG2	5.5e-05	2.3e-02	0.51	NDRG family member 2
NUDCD3	6.0e-05	2.4e-02	-0.33	NudC domain containing 3
FAM167B	7.6e-05	3.0e-02	0.76	family with sequence similarity 167 member B
PLK2	8.3e-05	3.2e-02	0.48	polo like kinase 2
TRIM15	9.1e-05	3.5e-02	0.42	tripartite motif containing 15
DLGAP1-AS1	1.0e-04	3.8e-02	0.49	DLGAP1 antisense RNA 1
KRTAP3-1	1.2e-04	4.2e-02	0.39	keratin associated protein 3-1
FXYD2	1.2e-04	4.2e-02	0.71	FXYD domain containing ion transport regulator 2
HOTAIRM1	1.3e-04	4.4e-02	1.12	HOXA transcript antisense RNA, myeloid-specific 1
GDF15	1.5e-04	5.0e-02	0.35	growth differentiation factor 15

Supplementary Table S2. STRING analysis revealed 19 interacting genes separated as a list of 9 upregulated and 10 downregulated genes.

gene	pval	padj	log2FC	description
RBM14	1.2e-38	1.9e-34	1.11	RNA binding motif protein 14
FXYD2	1.2e-04	4.2e-02	0.71	FXYD domain containing ion transport regulator 2
BTG2	9.1e-08	1.2e-04	0.64	BTG anti-proliferation factor 2
RBM4	5.1e-05	2.3e-02	0.62	RNA binding motif protein 4
PSPC1	5.7e-10	2.2e-06	0.58	paraspeckle component 1
PLK2	8.3e-05	3.2e-02	0.48	polo like kinase 2
RASD1	2.5e-05	1.4e-02	0.37	ras related dexamethasone induced 1
GDF15	1.5e-04	5.0e-02	0.35	growth differentiation factor 15
DDX17	1.2e-09	3.1e-06	0.32	DEAD-box helicase 17

gene	pval	padj	log2FC	description
NCL	3.7e-12	2.8e-08	-3.29	nucleolin
PFKP	1.2e-07	1.4e-04	-0.65	phosphofructokinase, platelet
PRKACB	4.0e-06	2.6e-03	-0.5	PRKACB divergent transcript
IDH2	7.6e-10	2.4e-06	-0.42	isocitrate dehydrogenase (NADP(+)) 2
FEN1	1.1e-07	1.3e-04	-0.38	flap structure-specific endonuclease 1
MYBL2	1.1e-05	6.6e-03	-0.38	MYB proto-oncogene like 2
MCM6	5.3e-05	2.3e-02	-0.35	minichromosome maintenance complex component 6
MSH6	4.7e-05	2.3e-02	-0.34	mutS homolog 6
CDC6	5.4e-05	2.3e-02	-0.34	coiled-coil domain containing 6
MCM3	2.4e-06	1.6e-03	-0.27	minichromosome maintenance complex component 3
